# The Role of Altered Nucleotide Excision Repair and UVB-Induced DNA Damage in Melanomagenesis

**DOI:** 10.3390/ijms14011132

**Published:** 2013-01-09

**Authors:** Timothy Budden, Nikola A. Bowden

**Affiliations:** Centre for Information Based Medicine, Hunter Medical Research Institute, and School of Biomedical Sciences & Pharmacy, Faculty of Health, University of Newcastle, Newcastle, NSW 2289, Australia; E-Mail: timothy.budden@uon.edu.au

**Keywords:** ultraviolet light, UVB, DNA damage, 6-4 photoproducts, cyclobutane pyrimidine dimers, DNA repair, nucleotide excision repair, cell death, apoptosis, melanoma

## Abstract

UVB radiation is the most mutagenic component of the UV spectrum that reaches the earth’s surface and causes the development of DNA damage in the form of cyclobutane pyrimidine dimers and 6-4 photoproducts. UV radiation usually results in cellular death, but if left unchecked, it can affect DNA integrity, cell and tissue homeostasis and cause mutations in oncogenes and tumour-suppressor genes. These mutations, if unrepaired, can lead to abnormal cell growth, increasing the risk of cancer development. Epidemiological data strongly associates UV exposure as a major factor in melanoma development, but the exact biological mechanisms involved in this process are yet to be fully elucidated. The nucleotide excision repair (NER) pathway is responsible for the repair of UV-induced lesions. Patients with the genetic disorder Xeroderma Pigmentosum have a mutation in one of eight NER genes associated with the XP complementation groups XP-A to XP-G and XP variant (XP-V). XP is characterized by diminished repair capacity, as well as a 1000-fold increase in the incidence of skin cancers, including melanoma. This has suggested a significant role for NER in melanoma development as a result of UVB exposure. This review discusses the current research surrounding UVB radiation and NER capacity and how further investigation of NER could elucidate the role of NER in avoiding UV-induced cellular death resulting in melanomagenesis.

## 1. Introduction

It has been long known that solar radiation is genotoxic, with UV radiation being the most mutagenic component [1]. UV-irradiation usually results in cellular death, but if left unchecked, it can affect DNA integrity, cell and tissue homeostasis and cause mutations in oncogenes and tumour-suppressor genes. These mutations, if unrepaired, can lead to abnormal cell growth, increasing the risk of cancer development [2,3]. Epidemiological data strongly associates sunlight exposure as a major factor in melanoma development [4–6], but the exact biological mechanisms involved in this process are yet to be fully elucidated.

**Figure 1:**
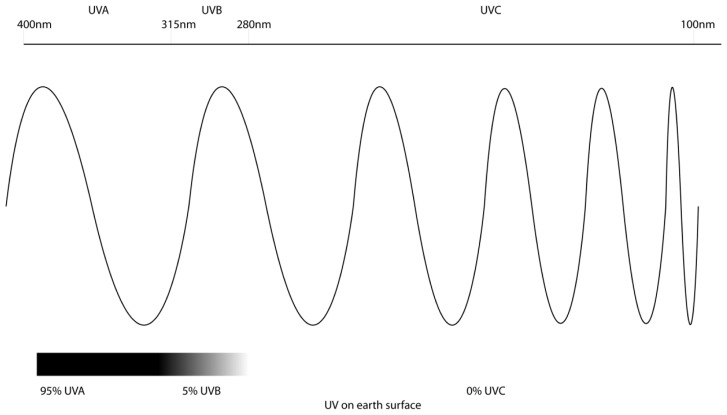
Ultraviolet radiation spectrum: Ultraviolet radiation is composed of three wavelengths. UVA has the largest wavelength (315–400 nm), making it the least energetic, but UV at the earth’s surface is composed of 95% UVA. UVB (280–315 nm) composes approximately 5% of the UV on earth surface. UVC is the smallest wavelength (100–280 nm) and also the most energetic and toxic. UVC is absorbed by the ozone layer, and none reaches the surface of the earth.

The UV spectrum is composed of UVC (100–280 nm), UVB (280–315 nm) and UVA (315–400 nm) (Figure 1). UVC has the shortest wavelength and is the most toxic, however, no UVC penetrates the ozone layer and reaches the earth’s surface [7]. The UV that does reach the surface of the earth is composed of approximately 95% UVA and 5% UVB, as much of UVB is also absorbed by the ozone layer [8]. Despite this, UVB remains the most mutagenic component of terrestrial UV, due to the fact that it is directly absorbed by DNA. While it is less abundant than UVA, it is absorbed by DNA more efficiently, with a tenfold difference between the two wavelengths. This results in UVB inducing more significant damage at much lower doses than UVA. It is currently debated whether UVA directly damages DNA or causes indirect oxidative damage. Therefore, it has been generally assumed that UVB is the predominant carcinogen in sunlight for melanoma development [1,9,10].

**Figure 2:**
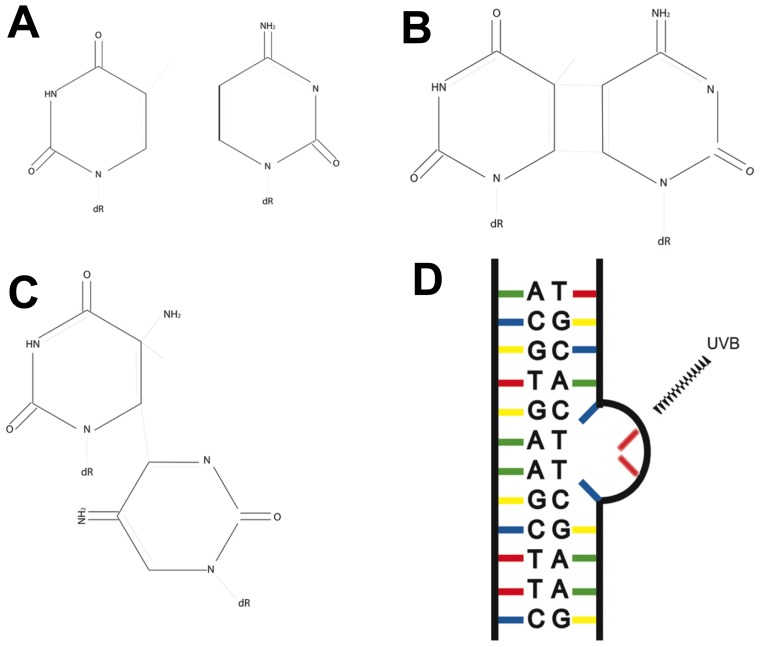
Structure of DNA photoproducts caused by UVB radiation: Cyclobutane pyrimidine dimers (CPD) and 6-4 Photoproducts (6-4 PPs) are the most common UV induced mutations. (**A**) Chemical structures of cytosine (C) and thymine (T); (**B**) The chemical structure of a TC CPD. UVB radiation reacts with the double carbon bonds in the adjacent thymine bases and creates additional covalent bonds; (**C**) The structure of TC 6-4 PP. A single covalent bond forms between a double carbon bond and a carbonyl group in adjacent pyrimidines, linking the two bases; (**D**) The additional bonds in both of these lesions result in a bulky adduct that affects the double helix structure of DNA, which can halt transcription and DNA replication (Image adapted from [2]).

The main photochemical reaction between UVB and DNA is absorption by pyrimidine bases, leading to the formation of two individual DNA photoproducts, cyclobutane pyrimidine dimers and 6-4 photoproducts (Figure 2). Photoproducts form at dipyrimidine sites (CC, CT, TT, TC), upon absorption of UVB. This predominantly occurs at sites containing a thymine, with TC and TT being more photoreactive. The resulting photoproduct creates a bulky lesion that distorts the DNA helix, creating adducts that can halt transcription and DNA replication (Figure 2) [11,12].

6-4 Photoproducts (6-4 PPs) are formed when UV reacts with the carbonyl group and double carbon bond in adjacent pyrimidines. This leads to an excited state and unstable oxetane, which is spontaneously rearranged to form a 6-4 PP [1]. These lesions are not believed to play a significant role in UV carcinogenesis, as they are removed with high efficiency [13]. Cyclobutane pyrimidine dimers (CPDs) are formed when UV induces the formation of additional covalent bonds between pyrimidine bases by reacting with double carbon bonds. The major type of CPD (TT) is formed approximately ten-times higher than that of the corresponding TT 6-4 PP, making CPDs the major photoproduct of UVB radiation. While the helix distortion created by 6-4 PPs is greater, CPDs are removed slower and are more mutagenic, being responsible for 80% of UVB mutations [1,14,15].

If unrepaired, UVB-induced DNA photoproducts can lead to mutations, most frequently C>T or CC>TT transitions. These are commonly referred to as UV fingerprint mutations, as they are the most specific mutation caused by UV, being rarely found in internal organs [16]. The exact mechanisms of how photoproducts lead to these UV fingerprint mutations are still unclear; however, it has been hypothesised that two pathways may exist. Mutations may be introduced when damaged DNA is replicated and translesion polymerase incorrectly replicates the pyrimidine base. Even though TT photoproducts are the most common, translesion polymerases, such as DNA polymerase η, can correctly replicate these bases, leading to a low mutation frequency [17,18]. The other pathway involves the deamination of 5-methylcytosine within CPDs leading to C>T transitions. Studies have shown that dipyrimidine sites containing 5-methylcytosine are 15-times more predisposed to CPD formation after UVB [19,20] and that deamination of these cytosines occurs at a significant rate [21]. Therefore, it is hypothesised that CPDs containing cytosine are susceptible to spontaneous deamination, which results in a C>T or CC>TT transition.

## 2. Ultraviolet Radiation and Melanoma

One hallmark feature of melanoma is development as a consequence of chronic UV exposure (reviewed in [22]). Both the incidence and mortality rates for melanoma are increasing rapidly worldwide. Incidence rates in Australia are amongst the highest in the world [23] and account for around 10% of all cancer cases in Australia [24]. It is the most common cancer in young Australians aged between 15 and 54 years [24]. Due to its aggressive nature and extreme resistance to chemotherapy treatments, there are very few effective treatments once melanoma has metastasised [25]. Therefore, survival for invasive melanoma is only 15%, making it a major clinical problem [26,27]. It is generally accepted that the two main factors in melanoma development are genetics and sun exposure [28]. However, while epidemiological data supports the undeniable causative link between melanoma and ultraviolet (UV) radiation, the exact molecular mechanisms are not completely understood [2,29].

## 3. Fingerprint Mutations Suggest a Role for UVB in Melanoma

UV fingerprint mutations are found across the genome of melanoma. Recently, a study by Pleasance *et al.* [30] sequenced the entire genome of a metastatic melanoma cell line, and a study by Berger *et al.* [31] sequenced the genome of 25 metastatic melanomas to compile a catalogue of somatic mutations. Additional studies have also sequenced the melanoma exome (protein coding regions of the genome) of up to 147 melanoma tumours [32–34]. All these studies identified that the vast majority of mutations present were C>T or CC>TT transitions. This mutational signature is indicative of UV-induced DNA damage, with C>T and CC>TT transitions being UV fingerprint mutations. Further investigation revealed that the majority of these substitutions occurred at dipyrimidines, sites at which photoproducts develop. The frequency of both these substitutions were elevated at CpG sites, which can be methylated to 5-methylcytosine, making them susceptible to CPD formation. The mutation spectrum in melanoma unequivocally demonstrates that the majority of somatic mutations are a result of UV-induced DNA damage; however, the role these mutations play in melanoma development is yet to be elucidated. Despite this, the most common somatic mutation found in melanoma, BRAF V600E (A>T), can be targeted with new therapies [35], but is not a UV fingerprint (C>T) mutation.

In addition to being found across the genome, UVB fingerprint mutations have also been found in key genes in all types of skin cancer. Interestingly, the most common mutations found in melanomas, in *BRAF* and *NRAS*, do not occur as a result of UV-irradiation. Basal cell carcinomas (BCCs) and squamous cell carcrinomas (SCCs) occur as a result of UV-exposure at a much higher frequency than melanomas, but rarely metastasize. This may be due in part to UV-induced mutations occurring in different genes in these skin cancer types. Mutations in the sonic hedgehog signalling genes PTCH or SMO are thought to be initiating mutations in >70% of BCCs [36]. SCCs, however, have characteristic large chromosomal aberrations and loss of heterozygosity (LOH), which underpins genomic instability [37,38]. Both BCCs and SCCs have high frequencies of UV-fingerprint mutations, particularly in *p53* [37,39,40] and *N-ras* [38].

*p53* is one of the most commonly mutated genes in cancer, which leads to alterations in cell cycle control and apoptosis [41]. While *p53* mutations are present in all types of skin cancer, they are less common in melanoma than in BCCs and SCCs [39]; *p53* has been shown to harbour UV signature C>T and CC>TT transitions in approximately 50% of all skin cancers [37,42], but has a higher rate of A>T in melanoma compared to other skin cancers [39], further implicating UV damage. The *p53* mutation hot spots are distinctly different in melanoma compared to BCCs and SCCs, which leads to the possibility that the pathways leading to a given tumour needs specific *p53* mutations [39].

Cyclin-dependent kinase inhibitor 2A (CDKN2A) is one of the best known genetic factors in melanoma development and codes for two proteins controlling cell proliferation: p16Ink4A and p14ARF [43]. A CDKN2A knockout mouse model has shown that melanoma develops after a single neonatal UV dose, suggesting that alterations in the CDKN2A may play an important role in the UV-induced development of melanoma [44]. A systemic review of studies on mutations in melanoma by Hocker *et al.* [45] showed that CDNK2A had statistically higher rates of UVB fingerprint mutations than other genes, such as *BRAF* and *NRAS* in melanoma, as well as compared to other non-skin cancers.

PTEN is a tumour suppressor gene that carries mutations in many human cancers and may also play a role in UV-induced DNA damage repair [46]. Studies have shown a mutation rate of approximately 30%–40% in melanoma cell lines [47,48]. A study examining mutations in PTEN in melanomas from patients with the skin cancer predisposing disorder Xeroderma Pigmentosum showed that of the melanomas analysed, 56% had mutations in PTEN, and of those, 91% were UVB fingerprint (C>T) mutations [49]. Additionally, 46% had more than one UVB mutation. This data provides further evidence for a role of UVB in the induction of melanoma. The evidence of UVB fingerprint mutations both across the melanoma genome and in specific genes that may be important in melanoma development or progression, including *p53*, *PTEN* and *CDNK2A*, highlights an important role for UVB in melanoma, strengthening the link between UV and melanoma.

The two most recent studies that have sequenced the melanoma exome by Krauthammer *et al.* [33] and Hodis *et al.* [32] both identified a novel mutation in the gene RAC1. This gene had a high rate of repeated mutation in 9.2% and 5% of melanomas, respectively, and carried a strong UV signature. RAC1 is a member of the Rho family of small GTPases and plays important roles in cell proliferation, cytoskeletal rearrangement, cell migration and adhesion [50–52]. The resulting amino acid change leads to stabilization of the active form of the protein, resulting in enhanced downstream signalling. Expression of the mutated form of RAC1 in melanocytes leads to enhanced MAP kinase signalling, as well as increased proliferation and migration, altering the melanocytes phenotype [33]. The data also indicated that this mutation is likely to occur during early mutagenesis, providing a possible link between this mutation, UV exposure and avoidance of cellular death.

## 4. The Nucleotide Excision Repair Pathway Removes UV-Induced Lesions

**Figure 3:**
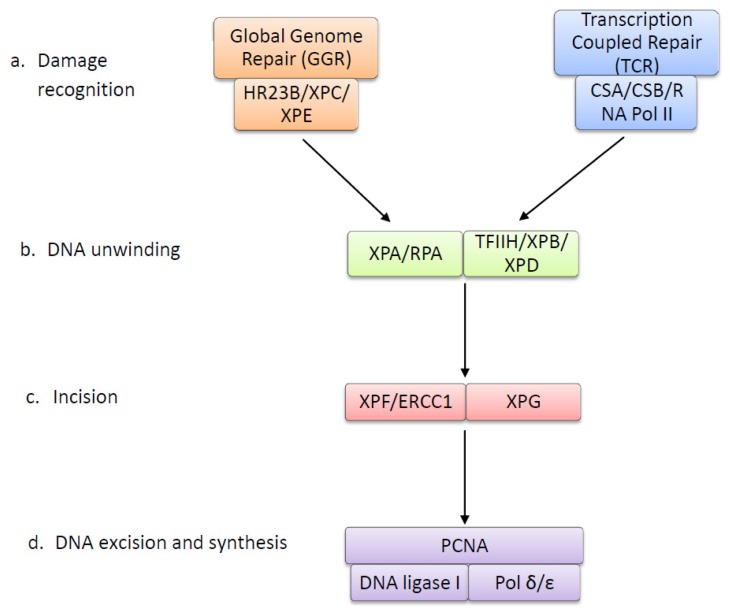
The nucleotide excision repair pathway: There are two primary damage recognition paths in nucleotide excision repair. Global genome repair (GGR) functions across the entire genome, including non-coding and non-transcribed genes. Transcription coupled repair (TCR) acts with higher priority to repair actively transcribed genes. In GGR, the XPE complex and XPC sense UV lesions and act to recruit repair proteins. In TCR, RNA polymerase II is stalled at the UV lesion and CSA and CSB recruit repair proteins. Following damage recognition, both pathways converge on a single repair path in which the TFIIH complex unwinds the DNA around the damage site, by means of helicases XPB and XPD. XPA binds to the site of DNA damage, while RPA binds to the undamaged DNA and allows for binding of endonucleases XPF-ERCC1 and XPG, respectively, which cleave and excise the damaged strand. Following this, DNA polymerase and PCNA synthesize new DNA to replace the damage. The process is complete when DNA ligase seals the nicks between the old and new DNA.

UV photoproducts alter the double helix structure of DNA by creating bulky adducts that halt transcription and trigger apoptosis (Reviewed in [53]), but if unrepaired, can result in mutations. In cells, DNA repair mechanisms are present that allow for damaged DNA to be removed and repaired, preserving the integrity of the genome. The nucleotide excision repair (NER) pathway is the system that is responsible for repairing bulky DNA damage, including UV-induced photoproducts, in particular CPDs and 6-4 PPs [54]. The NER pathway is a highly conserved system comprising around 30 proteins and functions in four main steps; damage recognition, DNA unwinding, excision and DNA synthesis [55]. The NER pathway is illustrated in Figure 3, and Table 1 summarises the function of each NER protein.

There are two sub-pathways in NER damage recognition, global genome repair (GGR) and transcription coupled repair (TCR). TCR repairs lesions that block transcription in actively transcribed genes. TCR functions with a higher priority than GGR and may work in concert with homologous recombination to efficiently repair damage [56]. It serves to rapidly recover transcriptional activity after DNA damage to prevent DNA-damage-induced apoptosis [57]. Induction of TCR is tightly linked to RNA polymerase II, which is stalled at the site of UV lesions and signals for repair with proteins CSA and CSB (reviewed in [58]). GGR is a slower repair process that functions across the entire genome, repairing silent, transcribed and non-transcribed regions. It is suggested to be the preferred pathway for removing 6-4 PPs and can remove these faster and more efficiently than CPDs [59]. In GGR, lesions are recognized by DNA-binding protein complexes XPC and XPE, which recognize and bind to UV lesions, signalling for repair. The main purpose of GGR is to remove lesions that may induce mutations during DNA replication, when translesion polymerases may introduce errors, thereby preventing carcinogenesis [60].

Following damage recognition, these pathways converge on a common repair pathway in which the first step is the unwinding of DNA around the damage by DNA helicases. The multi-protein complex TFIIH contains the helicases XPB and XPD and is recruited to the site of damage, unwinding the DNA around the site of the UV lesion to form a DNA bubble (reviewed in [61]). This allows for proteins XPA and RPA to bind to the DNA, XPA to the site of damage and RPA to the undamaged strand. These proteins allow for the binding of endonucleases XPG and XPF-ERCC1. XPG stabilizes the DNA bubble and cleaves the DNA 3′ to the UV-lesion. XPF-ERCC1 then cleaves the DNA 5′ to the lesion, and an oligonucleotide fragment containing the damage is removed. PCNA and DNA polymerase δ or ɛ then synthesise new DNA complementary to the undamaged strand. DNA ligase seals the nicks, completing the repair process [62].

The NER pathway has been linked to the tumour suppressor p53, particularly in the activation of the GGR pathway. P53 is a transcription factor that plays an important role in cell-cycle control, apoptosis and DNA damage response, with a critical role in protection against carcinogenesis [41]. One way that p53 may regulate DNA damage response is through its downstream target Gadd45, which plays a role in DNA repair, as Gadd45-deficient cells have reduced UV-induced DNA damage repair [84]. However, recent studies suggest that p53 may directly affect the expression of GGR genes *XPC* and *DDB2* (*XPE*). It has been shown that cells with mutations in p53 have deficiencies in the removal of both CPDs and 6-4 PPs, however this is limited to genomic DNA in non-transcribed regions, suggesting that it is GGR that requires functional p53 and that TCR removal of CPDs is still proficient [85,86]. One study has since shown that expression of *XPC* is UV-inducible in p53 wild-type cells; however, no induction is observed in p53 deficient cells. Further analysis of the gene showed a possible p53 response element, suggesting that p53 may directly control the expression of the *XPC* gene [87]. Another study has demonstrated that expression of *DDB2* (*XPE*) is also dependent on p53. It was observed that DDB2 mRNA levels was dependent on basal p53 expression and increased after DNA damage in a p53-dependent manner [88]. A subsequent study analysing the *DDB2* gene has shown that it also contains a region that binds p53 to control transcription [89]. Together, these results show that p53 directly controls DNA damage repair after UV damage through the expression of GGR genes *XPC* and *DDB2* (*XPE*) and that wild-type p53 expression is important for efficient GGR repair.

## 5. Xeroderma Pigmentosum: A Link between Nucleotide Excision Repair and Melanoma

The exact molecular mechanisms of the link between UV and melanoma are not completely understood, but a possible link is evident in the genetic disorder Xeroderma Pigmentosum (XP) in which the dysfunction of the NER pathway directly results in UV-induced skin cancers. XP is a rare autosomal recessive disease characterized by defective NER, extreme sun sensitivity, pigmentation abnormalities and an increased incidence of skin cancers, including melanoma [90]. Another genetic disorder, Cockayne syndrome (CS), is linked to the TCR component of the NER pathway and is caused by a mutation in CSA or CSB. However, unlike XP, there is no increase in cancer incidence, and all symptoms are neurological and developmental. Furthermore, no cases of cancer have been reported in CS patients [91]. Studies show that without functional TCR, the apoptotic response after UV is enhanced, with a significant induction of p53 and its target genes [92]. This suggests that after damage, the persistent blockage of RNA polymerase II tips the equilibrium of cell survival towards apoptosis, protecting from carcinogenesis [93]. As there is no increased incidence of cancer in CS patients as a result of TCR deficiency, it has been assumed that TCR would not play a role in melanoma development, as any disruption to TCR would lead to an enhanced apoptotic response, eliminating cancerous cells.

XP is a heterogeneous disease resulting from different mutations in one of seven NER genes or one DNA polymerase gene, resulting in eight different complementation groups, XP-A to XP-G and XP-V. Patients within each group will exhibit a large variability in symptoms and NER capacity depending on the nature of the mutation [94]. Groups XP-A and XP-C are the most prevalent (90% of cases) and are caused by mutation in the genes XPA and XPC, respectively. Only a few patients from other groups have been reported, most likely due to the biological importance of the enzymes that are mutated [95]. Most patients have a defect in both GGR and TCR, with the exception of patients from the XP-C and XP-E groups, who are only GGR-deficient. Patients from groups XP-A and XP-G are almost completely repair deficient, highlighting the critical role of XPA and XPG in the NER pathway. XP-B patients are very rare, due to the important roles of XPB outside the NER pathway, and XP-D and XP-F patients exhibit low levels of repair [96].

Mutation in the NER pathway and the subsequent reduced repair capacity results in XP patients having a high incidence of skin cancers compared to the normal population. This includes a 1000-fold increase in the incidence of melanoma [97,98]. There is a stronger association with cancer development in the complementation groups XP-C and XP-E, who have mutations in the GGR pathway [99]. XP-C is the most common group and results from a mutation in the XPC gene, leading to a repair capacity of 10%–20%, while XP-E patients, with a mutation in the DDB2 subunit of the XPE complex, show milder symptoms and have a repair capacity over 50%. Data from CS patients suggests that TCR capacity is the main determinant of cell survival after UV, supported by the fact that GGR deficient, but TCR proficient, XP-C and XP-E cells are less sensitive to UV-induced cell death. Therefore, as GGR is important in protecting from mutagenesis, it would be expected that low GGR capacity in XP-C and XP-E cells would to lead to an increase in mutation rates. This would then lead to an increased cancer predisposition, without cancerous cells being removed by UV-induced apoptosis [59,94,96,100]. Interestingly, most rodent cells are deficient in the repair of UV-induced CPDs in non-transcribed DNA and, hence, exhibit a defect in GGR [101]. Therefore, since rodents are used as surrogates for humans in environmental oncogenic studies, how they differ from humans with respect to GGR should be taken into consideration [102].

A relationship between UV mutagenesis and cancer development in XP patients is also seen when examining mutations. UV fingerprint mutations have been found in many genes in cancers from XP patients, including p53, CDKN2A and Ras oncogenes, many of which are also present in cutaneous melanomas that develop from sun exposure in the normal population [103–105].

In summary, hyper-mutability from UV-induced DNA damage and a higher incidence of melanoma in XP patients provides evidence of a relationship between reduced NER, UV-induced DNA damage and skin cancer development. Complementation groups XP-C and XP-E have impaired GGR and are more strongly associated with cancer development. This suggests that NER, in particular GGR, could play an important role in melanoma development as a result of UV exposure. If NER was compromised in melanocytes, then UV lesions would go unrepaired, leading to the accumulation of mutations and promotion of the transformation of melanoma. This could be a potential mechanism that links melanoma development to UV damage. Despite this possible connection between NER and melanoma and the recognized importance of DNA repair in melanomagenesis [106], very few studies have been performed to investigate the relationship between NER and UV damage in melanoma.

## 6. Research into NER and Melanoma

One area of increasing research is the investigation of single nucleotide polymorphisms (SNPs) in NER genes in predisposition to developing melanoma. Genetic variation that exists in DNA repair genes could lead to differences in repair capacity within the population, therefore predisposing to melanoma development. Several studies have investigated SNPs in NER genes, including XPC, XPG, XPD and XPF in case-control studies. The results of these studies are inconsistent and all studies have found different associations or no associations at all, often contradicting each other. Replication of results in any of these studies has not been achieved [107–109].

Recently, a Xiphophorus fish model of melanoma has been used by Fernandez *et al.* [110] to determine whether NER capacity plays a role in melanoma development. The Xiphophorus model develops melanoma, in part by overexpression and constitutive activation of fish paralogs of human *EGFR* and *CDNK2* (reviewed in [111]), but also after UVB exposure. This study used a hybrid model that results in reduced 6-4 PP removal to investigate the role of NER capacity. DNA was extracted from fish immediately and 24 hours after UVB exposure, and levels of 6-4 PPs were measured. It was reported that approximately 62% of 6-4 PPs were repaired after 24 h, with no significant difference in NER capacity between fish that developed melanoma and those that did not. It was also found that fish with 13.2% and 88.5% NER capacity both developed melanoma. From this data, the authors concluded that melanomagenesis is not associated with NER capacity. Some of the limitations of this study were the different DNA repair processes in Xiphophorus fish, the absence of quantitation of both 6-4PPs and CPDs, and the melanomas that develop in the Xiphophorus fish in this model have different histology to human melanomas [28]. Therefore, it cannot be concluded from this study that NER capacity does not play a role in the development of melanoma.

To date, there has only been one study that has compared UV-induced NER capacity in human melanocytes and melanoma cells. Gaddameedhi *et al.* [112] compared the repair of 6-4 PPs and CPDs between the two cell types to determine if NER plays a role in melanoma development. It was reported that there was a time-dependent reduction in photoproducts with 50%–80% of 6-4 PPs and 40%–80% CPDs being removed in both cell types. From this, it was concluded that there was no difference in the repair capacity of melanocytes and melanoma cells; therefore, melanoma cells retain normal NER function, which does not contribute to melanomagenesis. While it was concluded that there was no reduction in NER capacity, both cell lines showed high variation in repair capacity, ranging approximately 40%–80%. In addition, statistical analysis was not provided to prove that this reduction was not significant. The study was also not specific to measuring GGR or TCR, only the overall removal of photoproducts. Previous data has suggested that melanoma shows low levels of GGR, which would not have been identifiable in this study.

Melanocytes may initially have a lower NER capacity when compared with other cell types [113]. The repair of oxidative damage and photoproducts in melanocytes, normal and XP fibroblasts were compared, using host cell reactivation and *in vitro* repair synthesis assays. The ability of melanocytes to reactivate a UV damaged luciferase gene was lower than normal fibroblasts, but not as low as XP fibroblasts. Similarly melanocytes had a lower capacity of DNA damage-induced repair synthesis after UV irradiation compared to normal fibroblasts. It was also tested to see if the low repair was due to melanin in the melanocytes interfering with excision repair or expression of repair genes. Melanin was added to fibroblast lysates and showed a reduced repair capacity proportional to the amount of melanin. Additionally, it was found that melanin co-purified with DNA, and when separated after electrophoresis, the DNA became sensitive again to repair. This evidence suggests that melanin in melanocytes binds to DNA interfering with damage recognition and incision by NER enzymes, resulting in reduced repair. The results in this study may explain the low percentage of CPD and 6-4 PP removal in both melanocytes and melanoma cells reported by Gaddameedhi *et al.* [112]. Moreover, if melanocytes originally have a low repair capacity, then the lack of difference between melanocytes and melanoma cells cannot rule out NER playing a role melanoma development. Having a low NER capacity may predispose melanocytes to the accumulation of UV-induced DNA mutations that could lead to avoidance of cell death and melanomagenesis.

There are two hallmark features of melanoma; firstly, its development as a result of chronic UV exposure and, secondly, the limited efficacy of chemotherapy drugs, especially the DNA-damaging agent cisplatin [114]. Cisplatin induces intra- and inter-strand cross links in DNA that cause helix distorting lesions similar to UV damage. In addition to repairing UV-induced photoproducts, NER also removes cisplatin lesions [54]. A study by Bowden *et al.* [115] investigated the level of NER gene expression after cisplatin-induced DNA damage in melanoma and melanocytes. Results showed a higher basal expression of NER genes in melanoma; however, when compared to melanocytes, there was a significant lack of induction of GGR genes XPC, DDB1 and DDB2. In contrast, the expression of TCR genes CSA and CSB was low in both melanoma and melanocytes, suggesting that TCR does not play a role in the response to cisplatin. The findings of this study indicate that a lack of NER induction, especially GGR, may play a role in the resistance of melanoma to cisplatin. Subsequent studies identified that the NER component ERCC1 could play a key role in melanomagenesis and resistance to cisplatin [116,117]. In addition, the DNA damage caused by cisplatin is similar to that caused by UVB, with both causing helix distorting lesions. As a result, it could be hypothesized that melanoma would also have low expression of NER in response to UV damage similar to that seen in cisplatin. This could suggest a potential role for NER, particularly GGR, in the development of melanoma as a result of UV.

Furthermore, the genome of the melanoma cell lines sequenced by Pleasance *et al.* [30] and Berger *et al.* [31], which showed a mutational signature indicating DNA damage by UV exposure, also displayed an uneven distribution of mutations across the genome. There was a lower prevalence of UV mutations on the transcribed strand, as well as within exons compared to introns. This suggests that the TCR component of NER, which is responsible for removing damage from transcribed genes, is still functional in melanoma. Reduced GGR activity could also be responsible for the significant amount of UV mutations across the rest of the genome, as GGR removes UV lesions from the entire genome. These results are similar to those suggested by Bowden *et al.* [115]; that melanoma has low expression of GGR genes, but sufficient TCR expression and that low levels of the GGR component of NER may play a role in the development of melanoma. The idea that TCR does not play a role in melanoma development can be supported by the fact that Cockayne syndrome patients, who lack TCR, do not show an increased incidence of melanoma that is seen in XP patients.

If GGR was compromised in melanocytes, then UV-induced DNA damage across the genome would go unrepaired and lead to mutations when DNA is replicated. Additionally, cells would not undergo apoptosis to prevent cancer development, due to a functional TCR. This could then potentially lead to cell transformation and melanomagenesis, highlighting a potential role for NER in melanoma development.

## 7. Conclusions

The data suggests that the NER pathway may play a role in avoidance of UV-induced cell death and the subsequent development of melanoma. In particular the GGR pathway of NER may be important, as it functions to repair damage that can lead to mutations during DNA replication, and XP patients with defective GGR have a stronger association with cancer development. This idea has been supported by studies that found low GGR expression in melanoma cells treated with cisplatin and the genome of a melanoma line showing a high frequency of UV mutations in non-transcribed regions. Furthermore, melanocytes may potentially have low NER levels, which could predispose them to UV mutagenesis and transformation.

Despite this possible connection between NER and melanoma, little research in this area has been done, and the studies that have been performed are often contradictory. Given the scarce research into NER in melanoma, more investigation is needed to fully elucidate this relationship.

## Figures and Tables

**Table 1:** Components of the nucleotide excision repair pathway and their functions.

Protein	Function
XPC	DNA-binding protein that recognizes UV lesions in global genome repair and recruits subsequent repair proteins. Can easily detect 6-4 photoproducts (6-4 PPs), but requires XPE to recognize and bind to CPDs [63,64].
XPE	Composed of subunits DDB1 and DDB2. Detects and binds to both CPDs and 6-4 PPs via DDB2 subunit. Forms ubiquitin ligase complex, which polyubiquitinates XPC to increase affinity to UV damage, allowing recognition of CPDs [65,66].
HR23B	Forms a complex with XPC and prevents it from being degraded after polyubiquitination [67,68].
RNA Polymerase II	Stalled RNA polymerase II at the site of a UV lesion is the damage recognition step in transcription couples repair [69].
CSA (ERCC8)	Together with CSB, displaces stalled RNA polymerase and acts to recruit repair proteins [70].
CSB (ERCC6)	Together with CSA, displaces stalled RNA polymerase and acts to recruit repair proteins [70]. May ubiquitinate RNA polymerase II to enhance this process [71].
TFIIH Complex	Ten subunit protein ring complex, including XPB and XPD, that unwinds DNA around UV lesion to form denaturation bubble [72].
XPB (ERCC3)	DNA helicase subunit of TFIIH that unwinds the DNA damage site in the 3′ to 5′ direction [73].
XPD (ERCC2)	DNA helicase subunit of TFIIH that unwinds the DNA damage site in the 5′ to 3′ direction [73].
XPA	DNA damage verification by binding to the DNA damage that is marked by XPE and XPC and unwound by TFIIH. Allows for binding of XPF-ERCC1 complex [74].
RPA	Single-stranded DNA binding protein that binds to the undamaged strand opposite UV lesion and allows for binding of XPG [75,76].
XPG (ERCC5)	Endonuclease that makes the first incision 3′ to UV lesion [77].
XPF (ERCC4)	Forms an endonuclease complex with ERCC1 that makes the incision 5′ to UV lesion [78]. The XPF subunit of the XPF-ERCC1 complex contains the nuclease activity [79].
ERCC1	Forms an endonuclease complex with XPF that makes the incision 5′ to UV lesion [78]. ERCC1 subunit is required for binding to DNA [80].
DNA Polymerase δ/ɛ	Synthesises a new strand of DNA to replace the excised DNA containing the UV lesion [81].
PCNA	Required for DNA synthesis by acting with DNA polymerase δ/ɛ to form short repair patches [82].
DNA Ligase	Seals the nicks between the newly synthesised DNA strands [83].
